# Recent Advances in Dipeptidyl-Peptidase-4 Inhibition Therapy: Lessons from the Bench and Clinical Trials

**DOI:** 10.1155/2015/606031

**Published:** 2015-05-14

**Authors:** Jixin Zhong, Quan Gong, Aditya Goud, Srividya Srinivasamaharaj, Sanjay Rajagopalan

**Affiliations:** ^1^Department of Immunology, School of Medicine, Yangtze University, Jingzhou, Hubei 434023, China; ^2^Division of Cardiovascular Medicine, Department of Medicine, University of Maryland School of Medicine, Baltimore, MD 21201, USA

## Abstract

DPP4 inhibitors (DPP4i) are a class of newly developed antidiabetic drugs which preserve incretin hormones and promote postprandial insulin secretion. Although the cardiovascular effect of DPP4 inhibition has been substantially studied, the exact role of DPP4 in cardiovascular disease especially in humans remains elusive. Previous small studies and meta-analyses have suggested a benefit in both surrogate outcomes and cardiovascular events for these agents. However, there was growing evidence in recent years questioning the cardioprotective effect of DPP4i. Further, a signal of heart failure hospitalization in a recent large scale clinical trial SAVOR-TIMI 53 has called into question the safety of these agents and their utility in the treatment of cardiovascular disease. In this review, we will revisit the physiologic function of DPP4 and discuss its role in cardiometabolic disease based on recent experimental and clinical studies.

## 1. Introduction

Dipeptidyl-peptidase-4 (DPP4, also known as CD26) is a membrane glycoprotein that is well known for its role in the catalytic degradation of incretins. DPP4 inhibitors (DPP4i), as a class of antidiabetic medications, have been accepted worldwide, owing to their ease of administration, modest effects on HbA1c, and lack of serious side effects. DPP4 inhibition in experimental models has uniformly demonstrated cardioprotective effects. Indeed early meta-analyses of phase II/III data of DPP4i used in the context of glycemia lowering have shown favorable protective effects of this class in terms of cardiovascular (CV) endpoints, leading to a widespread expectation that these drugs will show a benefit in appropriately designed efficacy trials from a CV standpoint [1–3]. However, recently completed, appropriately designed, phase III trials with the intent of demonstrating benefit from a CV perspective have not shown significant improvement in primary CV endpoints in patients treated with DPP4i compared to placebo [4, 5]. In this review, we will summarize the structure and function of DPP4 and its known roles in physiology. We will also review its importance in the pathophysiology of cardiometabolic disorders and provide recent clinical trial evidence that has tested its effects in CV disease.

## 2. Overview of DPP4 Biology

**Figure 1:**
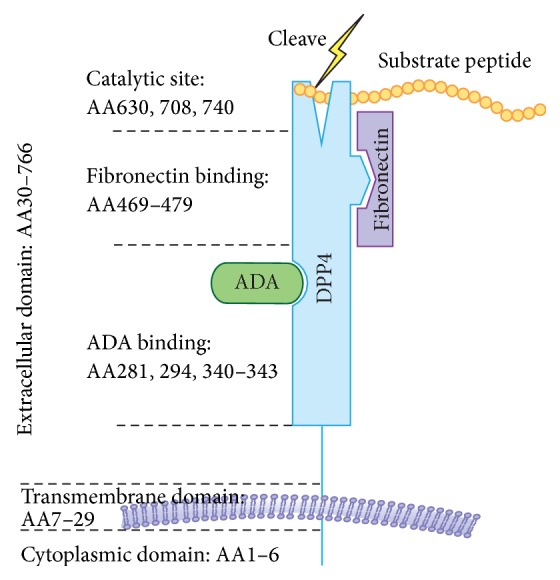
DPP4 molecular structure: DPP4 consists of a 6-amino-acid cytoplasmic tail, a 22-amino-acid transmembrane domain, and a large extracellular domain. The extracellular domain is responsible for the dipeptidyl-peptidase activity and binding to its ligands such as ADA and fibronectin. AA, amino acid; ADA, adenosine deaminase.

**Figure 2:**
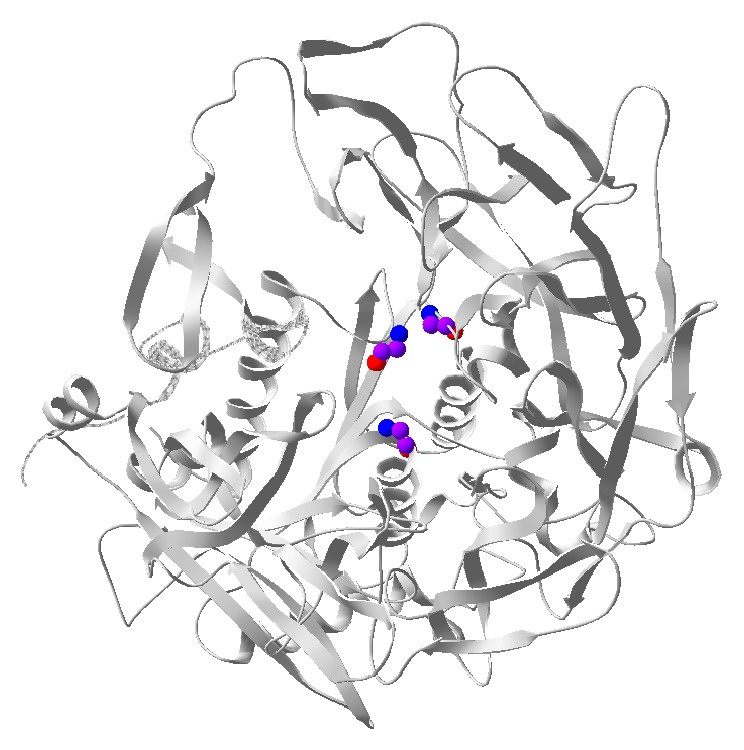
Catalytic triad of DPP4: backbone of residues that consist of DPP4 catalytic triad (Ser630, Asp708, and Hsp740) is shown (Purple: C; Blue: N; Red: O).

DPP4 is a transmembrane glycoprotein that forms a homodimer or tetramer on the plasma membrane and cleaves N-terminal dipeptides from proteins with proline or alanine as the penultimate (P1) amino acids. DPP4 is highly conserved among species in terms of amino acid sequence. As shown in Figure 1, DPP4 has a 6-amino-acid N-terminal cytoplasmic domain (AA1–6), a 22-residue transmembrane domain (AA7–29), and a large C-terminal extracellular domain. The extracellular component contains a *α*/*β*-hydrolase domain and an eight-blade *β*-propeller domain [6]. This domain is responsible for its dipeptidyl-peptidase activity and its binding to proteins such as adenosine deaminase (ADA) and fibronectin. Residue 294 and residues 340–343 within the cysteine-rich segment have been shown to be essential for ADA binding [7, 8], while residues 630, 708, and 740 are critical for the catalytic activity of DPP4 (Figure 2).

### 2.1. DPP4 Expression and Regulation

DPP4 is widely expressed in many organs, such as the kidney, spleen, lungs, pancreas, and prostate [9]. It is expressed at high levels on endothelial cells, differentiated epithelial cells, and some immune cells such as T cells, dendritic cells, and macrophages. DPP4 is also present in plasma as a soluble form, which either comes from a shedding process driven by proteinases or is released into circulation by means of vesicles such as exosomes, ectosomes, and apoptotic bodies. Altered expression of soluble DPP4 is commonly seen in many disorders such as solid tumors, autoimmune diseases, hepatitis C, type 2 diabetes (T2DM), and obesity [10–12].

The regulation of DPP4 expression is not fully understood. Studies have suggested that STAT1*α* [13] and hepatocyte nuclear factor-1 (HNF-1) [14] mediate the transcription of DPP4. In an in vitro experiment, cotransfection of HNF-1*α* and 1*β* enhanced reporter gene expression under the control of DPP4 promoter [14]. DPP4 promoter region also contains a GAS (interferon gamma-activated sequence) motif, which is a binding site of STAT1*α*. Indeed, STAT1*α* activation by administration of both interferons and retinoic acid leads to the binding of STAT1*α* to the GAS motif and a subsequent DPP4 transcription [13]. In addition to transcriptional regulation, DPP4 is also regulated at posttranscriptional level. IL-12 enhances the translation, but not transcription, of DPP4 in activated lymphocytes [15]. Many other cytokines are also involved in the regulation of DPP4 expression. IL-1*α* has been shown to be responsible for the upregulation of DPP4 in fibroblast, epithelial cells, and stromal cells [16, 17]. Polarization to T_H_17 by TGF*β*, IL-23, IL-6, IL-1*β*, and IL-21 also showed an increased expression of DPP4 [18].

### 2.2. Dipeptidyl-Peptidase Activity of DPP4

Incretin peptides such as gastric inhibitory polypeptide (GIP) and glucagon-like peptide (GLP-1) are responsible for the modulation of postprandial blood glucose by promoting insulin secretion from pancreatic *β* cells and via glucagonostatic effects. GLP-1 and GIP are rapidly inactivated by DPP4, leading to a short half-life (minutes for both GLP-1 and GIP). Mice lacking DPP4 (*Dpp4* ^−*/*−^) are protected from the development of diet-induced obesity and demonstrate improved postprandial glucose control [19, 20]. Pair-feeding and indirect calorimetry studies have shown that reduced food intake and increased energy expenditure accounted for the resistance to diet-induced obesity in* Dpp4* ^−*/*−^ mice.* Dpp4* ^−*/*−^ mice also demonstrate improved insulin sensitivity, reduced pancreatic islet hypertrophy, and protection against streptozotocin-induced *β* cell loss and hyperglycemia [19]. Pharmacological inhibition of DPP4 enzymatic activity improved glucose tolerance in wild-type but not in* Dpp4* ^−*/*−^ mice. DPP4 inhibition also improves glycemic control in* Glp1r* ^−*/*−^ mice, suggesting additional mechanisms for DPP4 inhibition-mediated antihyperglycemic effect [20].

In addition to incretin peptides, DPP4 also cleaves a number of other proteins. The physiologic targets include GLP1, GLP2, brain natriuretic peptide (BNP), peptide YY, stromal-cell-derived factor-1 (SDF-1), erythropoietin, granulocyte colony-stimulating factor (G-CSF), and substance P (3–11). Pharmacologic targets (evidence provided by in vitro cell culture and/or incubation experiments with substrate and DPP4) include gastrin-releasing peptide, growth-hormone-releasing factor, macrophage derived chemokine, eotaxin, IFN-*γ*-induced protein-10, granulocyte-macrophage colony-stimulating factor, erythropoietin, IL-3, neuropeptide Y, B-type natriuretic peptide, and peptide YY. The catalytic activity of DPP4 has been extensively reviewed elsewhere and will not be discussed here in detail [21–23].

### 2.3. Inflammation Mediated by DPP4-ADA Interaction

In addition to its peptidase activity, DPP4 also possesses noncatalytic function via interactions with a range of ligands including ADA, caveolin-1, fibronectin, coronavirus spike protein, collagen, glypican-3, insulin-like growth factor 2 receptor, fibroblast activation protein, and CXCR4. By interacting with these ligands, DPP4 plays a role in a variety of processes such as enhancing T cell activation and functional modulation of antigen presenting cells (APCs).

The costimulatory function of DPP4 was first described in early 1990s [24]. Ligation of DPP4 by ADA or anti-DPP4 antibodies recognizing ADA binding epitope enhanced T cell activation, proliferation, and cytokine production. Cross-linking by anti-DPP4 antibody induced tyrosine phosphorylation of a subset of proteins [25]. These phosphorylated molecules include signaling molecules downstream TCR/CD3, such as p56^1ck^, p59^fyn^, ZAP-70, MAP kinase, c-Cbl, and phospholipase C*γ* [26]. Since DPP4 has a very short intracellular domain (6 AAs), it relies on other proteins to transduce signaling in cells. Torimoto et al. reported that activation of DPP4 by its ligand leads to coaggregation of CD45RO into lipid rafts, suggesting that DPP4 may transduce costimulation via CD45 [27]. This result is consistent with the observation that DPP4 high T cells are restricted to the CD45RO^+^ cells [28] and CD4^+^ T cells lacking DPP4 cannot be triggered to elicit a memory T cell response [29]. As we will discuss below, DPP4-ADA interaction may also promote T cell activation by degrading adenosine, an immunosuppressive metabolite. In addition, interaction of DPP4 with caveolin-1 may form a complex consisting of DOO4, CARMA1, Bcl10, MALT1, and I*κ*B kinase in the lipid rafts on T cell membrane, leading to the activation of NF-*κ*B [30].

### 2.4. DPP4 as an Entry Protein for Coronavirus

Middle East Respiratory Syndrome (MERS), a viral respiratory illness, was first reported in Saudi Arabia in 2012. It was caused by the infection of a coronavirus, MERS-CoV. The mortality from MERS is approximately 30% [31]. DPP4 was subsequently identified as a functional receptor for the entry of MERS-CoV in human and bat cells [32]. The engagement of the MERS-CoV spike protein S with DPP4 mediates viral attachment and internalization. The residues involved in the DPP4 virus binding are identical to the ADA binding domain indicating a potential competition for DPP4 binding [33]. Human, macaque, horse, and rabbit DPP4 have been suggested to be able to bind MERS-CoV and therefore are susceptible to infection. However, small animals such as mice are more divergent with respect to the DPP4 virus binding region and are not susceptible to MERS-CoV infection [34]. DPP4 has been previously reported as a cofactor for the entry of HIV in the CD4^+^ T cells [35]. However, subsequent studies identified CCR5 and CXCR4 as the major coreceptors for HIV [36–39]. The coexpression of DPP4 and CCR5 may partially explain the association between DPP4 expression and HIV infection [40].

### 2.5. DPP4/Caveolin-1-Mediated Immune Activation

Ohnuma et al. reported that DPP4 interacts with caveolin-1 present on APCs and initiates a signaling cascade in antigen loaded APCs, resulting in their activation [41, 42]. Upon binding to DPP4, caveolin-1 is phosphorylated, resulting in the phosphorylation of IRAK-1 and its disassociation with Tollip. Phosphorylated IRAK-1 then activates NF-kappa B (NF*κ*B) [43, 44], which in turn upregulates CD86 [42]. The interaction between DPP4 and caveolin-1 has been reported to be involved in the pathogenesis of arthritis [45].

DPP4 has also been reported to bind multiple components of extracellular matrix such as collagen, fibronectin, and the HIV-1 Tat protein [46, 47]. Interactions with these matrix components may play a role in sequestration of DPP4 and allow additional functions such as matrix remodeling, metastasis, and chemotaxis.

## 3. DPP4 and Diabetes

### 3.1. DPP4 Expression in Obesity and Diabetes

Circulating DPP4 activity has been reported to be increased in patients with obesity and T2DM, positively correlating with HbA1c levels, degree of obesity, and measures of insulin resistance and inflammation [48, 49]. Lugari et al. reported that the increase of circulating DPP4 activity in diabetic patients results in a reduction of plasma GLP-1 (fasting and in response to meals) [50]. In addition to circulating DPP4 activity, the expression of DPP4 on T cells and dendritic cells is also increased in patients with T2DM [51]. However, there are also reports suggesting a decrease of circulating DPP4 activity in patients with T2DM [52]. This potential contradiction may relate to the fact that, in these studies, many patients were on concomitant medications. Several widely used antidiabetic medications including thiazolidinedione, pioglitazone, and metformin have been reported to reduce circulating DPP4 [53–55] and DPP4 expression on T cells [51]. This may reflect improvement in glycemic control and other measures of inflammation resulting in the reciprocal decrease in DPP4 expression. An enhanced expression of hepatic DPP4 has also been reported in nonalcoholic fatty liver disease and its expression may adversely affect glucose metabolism in this condition [56]. In vitro stimulation of HepG2 cells with high glucose increased the expression of DPP4, whereas insulin, fatty acids, and cholesterol did not [56].

### 3.2. Catalytic Function and Diabetes

Incretin hormones, GLP-1 and GIP, both potentiate insulin secretion from pancreatic *β* cells through G-protein-coupled receptors [57, 58]. As mentioned above, both GLP-1 and GIP can be inactivated by DPP4, resulting in a short half-life, less than 2 min for GLP-1 and less than 2 min in rodents or 7 min in human for GIP [59–61]. In patients with T2DM, incretin response is attenuated with an increase in plasma DPP4 enzymatic activity as well as heightened tissue DPP4 expression and release in tissues such as visceral adipose. The increase in DPP4 levels and expression correlates with the degree of glycemia/insulin resistance, suggesting that DPP4 mediated incretin degradation is involved in the pathogenesis of T2DM.

There are several DPP4i approved or being approved by FDA or EU as antidiabetic drugs, such as sitagliptin, saxagliptin, linagliptin, vildagliptin, and alogliptin. Most clinical trials with DPP4i demonstrate approximately a 0.6–0.8% lowering of HbA1C in patients with a baseline level around 8% [62]. The placebo-subtracted HbA1c reductions with DPP4i are generally greater in patients with higher baseline HbA1c. In general, most studies also corroborate improvements in homeostasis model assessment beta cell function index (HOMA-*β*) and fasting proinsulin : insulin ratio, suggesting improvement in *β* cell function [63]. The incidence of side effects and hypoglycemia are very low with these agents [63, 64]. In clinical trials, the most common reported side effects of DPP4i include nasopharyngitis, upper respiratory tract infection, urinary tract infection, and headache [65]. Saxagliptin [66] and linagliptin [67] were recently approved by FDA. In contrast to other members of the class, linagliptin has a primarily nonrenal route of excretion and therefore would not need dose adjustment, regardless of renal impairment [68]. In addition to those approved drugs, more DPP4i including dutogliptin and gemigliptin are under development and are awaiting FDA approval.

### 3.3. Noncatalytic Function and Diabetes

Inflammation plays a key pathogenic role in the development of insulin resistance and T2DM [69]. Innate immune mechanisms typified by macrophage infiltration and activation are widely believed to represent major mediators of adipose inflammation. However, recent findings suggest that T cells may also play an important role in this process. Activated CD8^+^ effector T cells have been shown to promote adipose inflammation by enhancing macrophage recruitment and activation, while CD4^+^ T cells especially T_H_2 and regulatory T cells which are the T cell subpopulations expressing lowest levels of DPP4 [18] were suggested to be protective in the development of adipose inflammation and insulin resistance [70]. As mentioned above, DPP4 provides T cell costimulatory signals. Therefore, DPP4 might play a role in the development of adipose inflammation and insulin resistance although there is a lack of direct evidence.

**Figure 3:**
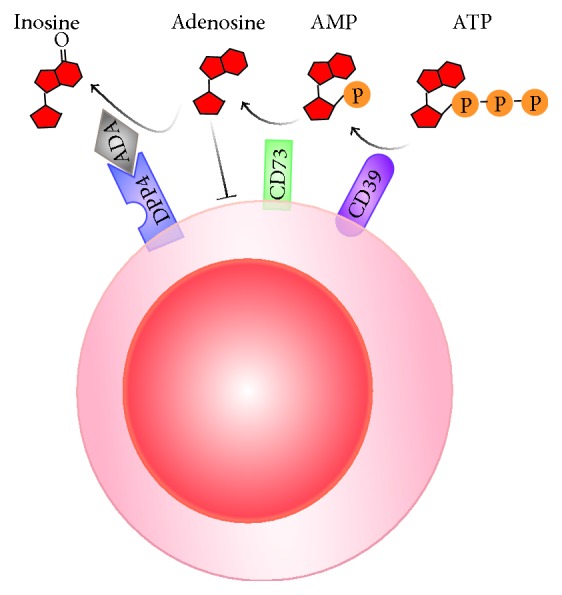
Role of ADA-DPP4 interaction in adenosine clearance: by binding to DPP4 on the cell surface, ADA reduces pericellular levels of cytotoxic metabolite adenosine by converting it into a nontoxic product inosine.

In addition to mediating costimulatory signaling, interaction between DPP4 and adenosine deaminase (ADA) may also facilitate T cell activation by providing a suitable microenvironment for T cell proliferation. Inherited mutations in ADA activity cause severe combined immunodeficiency (SCID) in both human and mice [71–73]. Extracellular ATP or ADP is initially converted to AMP by CD39 and CD73 to produce adenosine [74]. Adenosine is then processed by ADA and converted to inosine [75]. By anchoring ADA onto the cell surface, DPP4 modulates pericellular adenosine levels and thus regulates T cell activation (Figure 3). Absence of ADA activity results in the accumulation of adenosine, which dose-dependently inhibits T cell proliferation. Jurkat cells expressing DPP4 mutant devoid of ADA binding activity are sensitive to adenosine-mediated inhibition of T cell proliferation [7]. In contrast, cells expressing ADA and DPP4 on the surface are much more resistant to the inhibitory effect of adenosine [7, 76, 77]. We have recently demonstrated that DPP4 expressed on adipose tissue macrophages is involved in inflammation and insulin resistance by interacting with ADA [77]. Expression of DPP4 on adipose tissue macrophages was higher than that in circulation and was increased in obese and insulin resistant patients. Furthermore, DPP4 level on adipose tissue macrophage positively correlated with degree of insulin resistance. DPP4 on antigen presenting cells, including macrophages and dendritic cells, facilitated T cell proliferation and activation through its noncatalytic activity as catalytic inhibition of DPP4 or addition of exogenous sDPP4 did not affect their capability to stimulate T cells. Antigen presenting cell-expressing DPP4 was able to bind ADA and promote T cell activation via removal of suppressive effect of adenosine [77]. These results suggest that DPP4 on antigen presenting cells is capable of promoting inflammation and insulin resistance through its noncatalytic function. Interestingly, murine and rat DPP4 do not bind ADA [78]. Although adenovirus delivery of human DPP4 into mouse has been developed to study MERS-CoV infection [79], in vivo rodent models for the investigation of DPP4-ADA interaction of relevance to human diabetes are currently unavailable.

## 4. Role of DPP4 and Incretin System in Cardiovascular System

### 4.1. Incretins in Vascular Biology

The signaling of GLP-1 is mediated through GLP-1R. GLP-1R was originally identified in pancreatic *β* cells but is widely expressed in many tissues and organs including the lungs, kidney, central/peripheral nervous system, and the cardiovascular system [57]. The receptor for GLP-1 (GLP-1R) is expressed in cardiovascular cells including endothelial cells, cardiomyocytes, and coronary smooth muscle cells [80]. GLP-1R belongs to the family of G-protein-coupled receptors. The engagement of GLP-1R leads to the activation of adenylate cyclase through stimulatory Gs subunit and subsequent accumulation of cAMP in classically responsive cells such as pancreatic *β* cells [81, 82]. For example, in *β* cell, the activation of GLP-1 activates PKA, which subsequently reduces Foxo1 and results in an increase of Foxa2. Foxa2 then increases PDX-1, a transcription factor for insulin [83]. Via a cAMP-dependent pathway, GLP-1R signaling may also induce the activation of PI3K which further increases the expression of Bcl-2 and Bcl-xL, two antiapoptotic proteins [84].

There is evidence indicating that GLP-1 signaling is involved in the cardioprotective effects of DPP4 inhibition. For example, it has been shown that acute infusion of GLP-1 improves endothelial dysfunction in patients with T2DM [85, 86]. Exendin-4, a GLP-1R agonist, was also shown to stimulate proliferation of human coronary artery endothelial cells through eNOS-, PKA-, and PI3K/Akt-dependent pathways [87]. Both DPP4i and GLP-1 can increase endothelial progenitor cells (EPCs), suggesting a role of a GLP-1-dependent pathway in the development of EPCs [88, 89]. Moreover, GLP-1 can increase left ventricular developed pressure and coronary flow in isolated mouse hearts [90]. Further studies have confirmed that the protective effect of GLP-1 on endothelial cells is mediated through increasing nitric oxide (NO) production [91]. Activation of GLP-1R appears to have cardioprotective effect in humans and various animal models and its effects on contractility, blood pressure, and cardiac output appear to be independent of its antidiabetic effect [92]. Both enzymatic inhibition and genetic deletion of DPP4 reduced heart infarct size in ischemic models by preserving GLP-1 [1, 93]. In vitro treatment of cardiomyocyte with GLP-1R agonist reduced caspase-3 cleavage and apoptosis induced by various stimulations such as TNF*α*, hypoxia, and lipidemia [94–96]. Zhao et al. reported that GLP-1 increased p38 MAPK activity, nitric oxide (NO) production, and GLUT1 expression in isolated hearts and thus increased myocardial glucose uptake during aerobic perfusion [97]. Consistent with this, Nikolaidis et al. reported that 48 h continuous GLP-1 infusion (1.5 pmol/kg/min) in a conscious dog increased myocardial glucose uptake and left ventricular hemodynamics [98]. Pretreatment of p38 MAPK inhibitors could inhibit this effect [99]. However, there have been conflicting observations of GLP-1 effect on myocardial contractility. GLP-1 was reported to be able to decrease contractility in primary cultured adult rat cardiomyocytes, despite increasing cAMP levels [100]. The addition of 0.5 nM GLP-1 to the perfusate reduced left ventricular developed pressure [97], while 0.3 nM GLP-1 increased left ventricular developed pressure by 20% during Langendorff aerobic perfusion of isolated mouse hearts [90]. Studies on* Glp1r* ^−*/*−^ mice suggest that the absence of GLP-1R increases baseline left ventricular developed pressure [90]. Although acute administration of GLP-1 or exendin-4 increases heart rate and blood pressure at least in rodent models [101–103], chronic treatment of exendin-4 (20 nmol/kg twice daily) for 12 wk displayed a marked reduction in systolic blood pressure in both db/db mice and angiotensin-II-infused C57BL/6 mice [104]. In line with these animal studies, majority of clinical trials have also reported a reduction of blood pressure after chronic treatment of GLP-1R agonist [105–107]. It has also been suggested that GLP-1 exerts protective effect on atherosclerosis. Continuous infusion of exendin-4 for 4 weeks reduced neointimal formation [108], foam cell formation [109], and atherosclerotic lesion size [109, 110]. Some of these effects may relate to favorable effects of GLP-1 on lipoprotein metabolism. Infusion of GLP-1 (20 pmol/kg/min) via jugular vein reduces triacylglycerol absorption and intestinal ApoB-48 production in rats [111]. Hsieh et al. reported that exendin-4 (24 nmol/kg, i.p.) and sitagliptin (10 mg/kg, gavage) reduced postprandial triacylglycerol and ApoB-48 after oral fat load in C57BL/6 mice, whereas* Glp1r* ^−*/*−^ mice had an increased plasma level of triacylglycerol after oral fat load [112]. Human studies have shown that acute GLP-1 infusion (1.2 pmol/kg/min) suppressed postprandial plasma triacylglycerol and free fatty acid levels [113]. Short-term treatment of GLP-1 (25 nmol sc injection before meals for 5 days) decreased plasma VLDL-triacylglycerol by 38% [114]. The lipid lowering effects of GLP-1 and DPP4 have been reviewed in detail previously [115].

### 4.2. DPP4 in Vascular Biology

DPP4 is expressed in endothelial cells and may mediate cardiovascular effects in both GLP-1-dependent or -independent manner, although the functional significance of these effects has not been fully elucidated. By incubating isolated aorta rings with DPP4 enzymatic inhibitor, we provided direct evidence showing that DPP4 inhibition relaxes aorta through a GLP-1-independent pathway. Alogliptin, a DPP4 inhibitor, but not GLP-1, induces eNOS and Akt phosphorylation (Ser^1177^ and Ser^473^, resp.) paralleled by a rapid increase in nitric oxide [116]. Inhibition of Src kinase decreased eNOS and Akt phosphorylation in response to alogliptin, in contrast to a lack of any effect on insulin mediated activation of the eNOS-Akt, suggesting that alogliptin mediates vasodilation through Src kinase mediated effects on eNOS-Akt. DPP4 has also been shown to assist in angiogenesis by promoting EPCs in a GLP-1-independent pathway. Stromal derived factor-1*α* (SDF-1*α*), a known substrate of DPP4, is a regulator of EPCs. By comparing four weeks of sitagliptin versus no additional treatment added to baseline metformin and/or sulfonylurea therapy in 32 diabetic patients, Fadini and coworkers demonstrated that patients with DPP4 inhibition had a 2-fold increase of EPCs and a 50% increase of SDF-1*α* [117].

DPP4 may also affect cardiovascular function through regulation of inflammation. DPP4 is highly expressed on many inflammatory cells such as T cells and regulates their biological function [49]. Both enzymatic and nonenzymatic functions of DPP4 play an important role in T cell activation. T cell activation can be blocked by enzymatic inhibition of DPP4 [118, 119]. Suppression of dipeptidyl-peptidase activity of DPP4 resulted in a reduced production of cytokines including IL-2, IL-10, IL-12, and IFN-*γ* by peripheral blood mononuclear cells and T cells [118, 120, 121]. TGF-*β*1, an immunosuppressive cytokine, was shown to be upregulated by DPP4 inhibition [121, 122]. T cells transfected with mutant DPP4 devoid of enzymatic activity displayed reduced activation compared to those cells transfected with wild-type DPP4 [123]. Consistent with this finding, the addition of soluble DPP4 promoted the recall antigen-induced proliferation of peripheral blood lymphocytes, while soluble DPP4 mutant without enzymatic activity did not, providing a direct evidence that enzymatic activity of DPP4 is involved in the T cell activation [124]. As mentioned previously, DPP4 also promotes inflammation through catalytic independent mechanisms such as interactions with ADA and caveolin-1 [47]. However, there were also reports indicating that DPP4 may negatively regulate immune response. Mice deficient for DPP4 showed enhanced severity of autoimmune encephalomyelitis and reduced TGF*β* and increased production of IFN*γ* and TNF*α* when immunized with myelin oligodendrocyte glycoprotein. Inhibition of DPP4 activity has also been shown to enhance hematopoiesis by preserving colony-stimulating factor activity [125–127]. Those immunosuppressive functions may at least in part explain the lack of improvement in cardiovascular endpoints on T2DM patients with DPP4i.

### 4.3. Role of DPP4 in Cardiovascular Disease

There is good evidence that DPP4 inhibition mediates protective effect on myocardial infarction, hypertension, and atherosclerosis. Survival rate and infarct size significantly improved in* Dpp4* ^−*/*−^ mice after LAD ligation, accompanied by enhanced prosurvival signal pathways, such as pAKT, pGSK3*β*, and atrial natriuretic peptide in cardiac tissue [1]. Pharmacologic inhibition with sitagliptin also enhances expression of cardioprotective proteins and improved functional recovery after I/R injury in the murine heart [1]. Several studies in animal models support a favorable effect of DPP4 inhibition in improving endothelial function and blood pressure [116, 128, 129]. Saxagliptin treatment, for instance, has been shown to reduce blood pressure in the spontaneously hypertensive rat, an effect that was accompanied by an increase in aortic and glomerular NO release with comparable reductions in peroxynitrite levels [130]. In contrast to the positive effects in animal experiments, the effects of DPP4i on endothelial function in humans have not always been positive. In a double blind study in T2DM, treatment with vildagliptin for 4 weeks improved forearm blood flow in response to intra-arterially delivered acetylcholine [131]. In contrast, 2 studies using flow mediated dilation (FMD) of the brachial artery have shown diametrically opposite results. In one study, 2 DPP4i (sitagliptin and alogliptin) actually appeared to worsen FMD when used to treat T2DM [132]. In another study, sitagliptin improved FMD in association with an increase in CD34^+^ cells [133].

Both GLP-1 agonism and DPP4 inhibition reduce postprandial triacylglycerol and ApoB-48 in rodents [111, 112]. Human studies have shown that GLP-1 infusion (1.2 pmol/kg/min) for hours suppresses postprandial plasma triacylglycerol and free fatty acid levels [113]. A short-term treatment of GLP-1 (25 nmol sc injection before meals for 5 days) decreased plasma VLDL-triacylglycerol by 38% [114]. Continuous infusion of exendin-4 for 4 weeks reduces neointimal formation [108], foam cell formation [109], and atherosclerotic lesion size [109, 110] in mice. By providing 12 weeks of alogliptin treatment, Shah et al. demonstrated that DPP4 inhibition reduced atherosclerotic plaque and vascular inflammation in atherosclerosis prone* Ldlr* ^−*/*−^ and* ApoE* ^−*/*−^ mice [134]. However, human studies reveal contradictory results on the cardiovascular effects of DPP4 inhibitors. Patil et al. reported in a recent meta-analysis including 18 randomized clinical trials and 4,988 patients on DPP4 inhibition therapy and 3,546 patients on control treatment (other diabetic treatments or placebo) and demonstrated that DPP4 inhibitors are safe from a cardiovascular standpoint and have beneficial effects on cardiovascular events compared to other diabetic medications and placebo [3]. Another meta-analysis with 70 trials and 41,959 patients performed by Monami et al. also suggested that DPP4 inhibition reduced cardiovascular risk and all-cause mortality in diabetic patients [135]. In contrast, a more recent meta-analysis of 50 trials with 55,141 participants reported that DPP4 inhibitors have no difference in all-cause mortality, CV mortality, acute coronary syndrome, or stroke compared with placebo. Furthermore, DPP4 inhibitors showed a statistically significant increase in heart failure outcomes (RR = 1.16; 95% CI 1.01–1.33; *P* = 0.04) [136]. The association between DPP4 inhibition and increased risk of heart failure is also supported by another meta-analysis by Monami in 2014 [137]. More importantly, the results from two large scale clinical trials completed recently also did not support the cardioprotective effect of DPP4 inhibition in humans.

### 4.4. Results from Large Scale Clinical Trials of DPP4 Inhibitors

There are several phase-3 or phase-4 clinical trials assessing the cardiovascular effect of DPP4i. These trials include EXAMINE (EXamination of cArdiovascular outcoMes with alogliptIN versus standard of carE in patients with T2DM and acute coronary syndrome) on alogliptin and SAVOR-TIMI 53 (Saxagliptin Assessment of Vascular Outcomes Recorded in Patients With Diabetes Mellitus-Thrombolysis in Myocardial Infarction 53 trial) which have been completed. The ongoing studies include TECOS (ClinicalTrials.gov Identifier: NCT00790205) on sitagliptin, CAROLINA (ClinicalTrials.gov Identifier: NCT01243424) on linagliptin, CARMELINA (ClinicalTrials.gov Identifier: NCT01897532) on linagliptin, MK-3102-015 AM1 (ClinicalTrials.gov Identifier: NCT01697592) on omarigliptin (MK3102), and MK-3102-018 (ClinicalTrials.gov Identifier: NCT01703208) on omarigliptin.

In the EXAMINE study, 5,380 diabetic patients with a recent (<90 days) myocardial infarction or unstable angina requiring hospitalization were randomized to the treatment of alogliptin or placebo. The patients were followed for a median period of 18 months. Although a safety trial was not designed to answer questions on efficacy, alogliptin treatment did not improve the combined outcome of primary endpoints including death from cardiovascular causes, nonfatal myocardial infarction, and nonfatal stroke [4]. There was a trend towards reduction in cardiovascular death albeit nonstatistically significant. A total of 16,492 subjects with HbA1c 6.5-12% and one of the following cardiovascular risks: a history of MI, atherosclerosis, hypertension, smoking, or dyslipidemia, were recruited in SAVOR-TIMI 53 to evaluate the effect of saxagliptin on cardiovascular outcomes. The patients were randomized to saxagliptin or placebo group. Additional antidiabetic agents were prescribed throughout the study. Median follow-up time was 2.1 years and maximum was 2.9 years. In consistency with the results from EXAMINE, no improvements in cardiovascular outcomes were observed in saxagliptin treatment group compared to placebo-treated patients. It is noteworthy that the hospitalization rate for heart failure was higher in saxagliptin-treated subjects (3.5% versus 2.8%; hazard ratio, 1.27; 95% CI, 1.07 to 1.51; *P* = 0.007) [5]. No differences in multiple other safety endpoints (such as pancreatitis, cancer, and hypoglycemia) were observed across groups in both SAVOR-TIMI and EXAMINE trials. These trials suggest that catalytic inhibition of DPP4 is basically safe from a cardiovascular standpoint but also does not improve cardiovascular endpoints at least in the short term. It must be noted that the median follow-up period for EXAMINE and SAVOR-TIMI 53 was 1.5 years and 2.1 years, respectively, and a longer follow-up period may be necessary to further confirm the results. At least from the results of current large scale trials, it is suggested that catalytic inhibition alone is insufficient to improve cardiovascular outcomes. The other ongoing trials assessing the cardiovascular safety of DPP4i may provide further insights into the cardiovascular effect of DPP4.

### 4.5. Effect of DPP4 Inhibition on Heart Failure

Animal studies have suggested that DPP4 inhibition improved cardiac function and survival rate of heart failure. Gomez et al. reported that DPP4 activity was positively associated with body weight in adult dog and was significantly higher in heart failure class 1 compared with healthy heart and heart failure class 3, demonstrating that DPP4 might be involved in the early stage of heart failure [138]. In a pressure-overload-induced heart failure animal model (transverse aortic constriction, TAC), Takahashi reported that vildagliptin ameliorated TAC-induced left ventricular enlargement and dysfunction and improved survival rate on day 28 (TAC with vildagliptin, 67.5%; TAC without vildagliptin, 41.5%; *P* < 0.05). In accordance with this, Shigeta et al. demonstrated that both pharmacological and genetic DPP4 inhibition reversed diabetic diastolic left ventricular dysfunction and pressure-overload-induced left ventricular dysfunction [2].

Improved left ventricular function assessed by ejection fraction, mitral annular systolic velocity, and peak systolic velocity was also observed in humans with DPP4 inhibition (sitagliptin, single dose or 4 weeks of treatment) [34, 40]. GLP-1 agonists have also been shown to increase left ventricular ejection fraction [139–141] (all in short term), although there have been inconsistencies [142, 143]. Despite the potential benefits in improving cardiac function from short-term use of DPP4i and GLP-1 analogs, reports on long-term cardiovascular effect of DPP4i and GLP-1 analogs are limited. Surprisingly, Scirica et al. reported an increased hospital admission rate for heart failure in saxagliptin-treated patients compared with placebo (3.5% versus 2.8%; hazard ratio, 1.27; 95% CI, 1.07 to 1.51; *P* = 0.007) in SAVOR-TIMI 53 trial [5], raising the question whether DPP4i increase the risk for heart failure [144]. In a subsequent meta-analysis including 84 trials and 69,615 patients, Monami et al. reported that DPP4i (including vildagliptin, sitagliptin, saxagliptin, alogliptin, linagliptin, and dutogliptin) increased the overall risk for heart failure with an OR of 1.19 (95% CI: 1.03 to 1.37; *P* = 0.015) although these results were heavily influenced by the SAVOR-TIMI 53 results [137]. Interestingly, another meta-analysis which pooled 20 randomized controlled studies and 9,156 patients to assess the cardiovascular safety of saxagliptin reported that saxagliptin did not increase the incidence rates for cardiovascular events including heart failure, with an incidence rate ratio of 0.55 (95% CI: 0.27 to 1.12) for heart failure [145]. In a study published July 2014, Weir et al. further evaluated the effect of sitagliptin (Januvia) on cardiovascular endpoints (all-cause hospital admission/death as well as heart-failure-specific hospitalization/death in 7,620 diabetic patients recently diagnosed with heart failure) [146]. Although sitagliptin treatment did not increase the risk for primary endpoints or each component (hospital admission or death), it was noted to be associated with increased risk for heart failure hospitalization (12.5% versus 9.0%; OR: 1.84; 95% CI: 1.16–2.92). The absolute risks associated with DPP4i for heart failure are small. One limitation of this meta-analysis should not be overlooked with the principal criticism being that they include studies that were never designed for cardiovascular signals [147].

Are there risk factors that may identify patients at risk for this small risk seen with saxagliptin? In the SAVOR-TIMI 53 trial, Scirica et al. reported that patients with increased risk for hospitalization for heart failure had either prior heart failure or elevated levels of natriuretic peptides or chronic kidney disease. However, the risks of the primary and secondary endpoints were similar between treatment groups even in patients at high risk for hospitalization for heart failure [148], suggesting that DPP4 inhibition by saxagliptin does not increase death, myocardial infarction, or stroke even in those with increased risk for heart failure [149]. Therefore, these recent findings may not limit the use of DPP4i in T2DM patients with cardiovascular disease. However, DPP4i should be used with caution in T2DM patients with increased vulnerability to DPP4 inhibition-associated heart failure, especially with saxagliptin such as history of heart failure or chronic kidney disease. The ongoing studies such as TECOS, CARMELINA, and CAROLINA should be informative of additional risks posed by these agents as a class.

## 5. Safety of DPP4i

Early studies on the safety suggested a well tolerability of DPP4i with only some minor side effects such as gastrointestinal reaction (nausea, vomiting, diarrhea, etc.), flu-like symptoms, and skin reactions [63, 150, 151]. More importantly, they showed no weight gain and hypoglycemic risk compared to other oral antidiabetic medications [152]. Considering the diverse functions of DPP4, the safety of long-term usage of DPP4i on immune function such as antitumor and anti-infection was questioned [153]. An altered expression level of DPP4 has been reported in many types of cancer [153]. An increase in pancreatic cancer was noted in patients taking sitagliptin or exenatide compared with other therapies as evidenced by an evaluation of US FDA adverse event database [154]. But the incidence of all other cancers was similar between sitagliptin group and control drugs [154]. Another evaluation of the German adverse event database, in contrast, showed no significant association between pancreatic cancer and DPP4i [155]. Several meta-analysis reports indicate an increased risk of infection such as nasopharyngitis and urinary tract infection in patients on DPP4i [156, 157]. However, there were also contradictory reports on infections. Monami et al. reported in a meta-analysis that there was a significant increase in the risk of nasopharyngitis with sitagliptin (Mantel-Haenszel odds ratio (MH-OR): 1.43; 95% CI: 1.07–1.91; *P* = 0.017), but not with vildagliptin. No significant associations with upper respiratory tract infection and urinary tract infection were observed. The actual incidence of other infections was lower than in comparator groups [152].

There were also some studies raising concerns that incretin-based therapy may potentially contribute to pancreatitis [158, 159]. However, there are very limited clinical data examining the risk of DPP4i on pancreatitis and the conclusion remains uncertain [160]. A meta-analysis including 134 trials on DPP4i and pancreatitis risk showed no significant increase of pancreatitis with DPP4i (MH-OR: 0.93; 95% CI: 0.51–1.69; *P* = 0.82) [161]. It is noteworthy that the number of observed cases was small and further investigations are needed to confirm the result. Elashoff et al. reported a 6-fold increase of pancreatitis in patients treated with sitagliptin compared with patients treated with control antidiabetic drugs [154]. In contrast, a recent case-control study including 12,868 patients' first-time hospitalization for acute pancreatitis from 2005 to 2012 also reveals no associations between DPP4i and acute pancreatitis [162].

## 6. Conclusion

The development and application of specific DPP4i in clinic reemphasized the importance of DPP4 in both physiological and pathological processes. Recent studies suggest that DPP4i are safe from cardiovascular perspective although no significant improvement was observed in cardiovascular endpoints on T2DM patients with antidiabetic medications. However, it must be acknowledged that the durations of these two trials are less than 5 years and all subjects were on medications such as statins which may interfere with the cardiovascular effects of DPP4i. Notably, at least one trial showed an increase of heart failure hospitalization in T2DM patients with prior heart failure or chronic kidney disease, suggesting that DPP4i should be used in caution in those patients. The absolute risks for heart failure agents are small with these agents and it must be acknowledged that not only diabetes but other therapies used for glycemia control such as sulfonylureas and thiazolidinediones also increase the risk for heart failure. Further studies are required to investigate the exact role of DPP4i, particularly their catalytic independent activity in cardiovascular disease.
